# Evaluation of the effect of the PRECEDE PROCEED model-based education program on hemodialysis patients’ treatment compliance, healthy lifestyle behaviors, and quality of life

**DOI:** 10.1097/MD.0000000000049759

**Published:** 2026-07-10

**Authors:** Ayşenur Sariaslan, Mehtap Kavurmaci

**Affiliations:** a Department of Internal Medical Nursing, Kafkas University Faculty of Health Sciences, Kars, Turkey; b Department of Internal Medical Nursing, Ataturk University Faculty of Nursing, Erzurum, Turkey.

**Keywords:** healthy lifestyle behaviors, hemodialysis, nursing, PRECEDE–PROCEED model, quality of life, treatment adherence

## Abstract

**Background:**

Improving health-promoting lifestyle behaviors, treatment adherence, and quality of life is important for patients undergoing hemodialysis. This study aimed to evaluate the effects of a PRECEDE–PROCEED model (PPM)-based educational program on health-promoting lifestyle behaviors, treatment adherence, and quality of life among patients undergoing hemodialysis.

**Methods:**

This study employed a controlled pretest–posttest–follow-up design to evaluate a community-based educational program developed using the PPM. Patients were assigned to either an intervention group (n = 34) or a control group (n = 32) according to their hemodialysis treatment schedules to minimize contamination between groups. The primary outcome was health-promoting lifestyle behaviors measured using the healthy lifestyle behaviors scale II (HLBS-II). Secondary outcomes included treatment adherence and quality of life. Data were collected using the MARS, FCHPS, SDBHP, HLBS-II, and SF-12 scales and analyzed using linear mixed-effects models.

**Results:**

Significant group × time interaction effects were identified for HLBS-II total scores, medication adherence, dietary behaviors, fluid control behaviors, and quality-of-life scores in favor of the intervention group (*P* < .05). The intervention group demonstrated significant improvements in health-promoting lifestyle behaviors, particularly in health responsibility, physical activity, stress management, and interpersonal relations. Improvements in both physical and mental quality-of-life scores were also observed following the intervention.

**Conclusion:**

The findings suggest that a PPM-based educational program may improve health-promoting lifestyle behaviors, treatment adherence, and quality of life among patients undergoing hemodialysis. These results support the use of theory-based educational interventions in hemodialysis care.

## 1. Introduction

Chronic kidney disease (CKD) is a major and growing global health problem. It is defined by the presence of an estimated glomerular filtration rate < 60 mL/min/1.73 m^2^ for at least 3 months, an albumin-to-creatinine ratio ≥ 30 mg/g, or other markers of kidney damage, regardless of the underlying cause. In individuals with CKD, impaired kidney function necessitates treatment, such as kidney transplantation or renal replacement therapy, to prevent progression to kidney failure.^[1]^

Hemodialysis (HD) is one of the most commonly used renal replacement therapy modalities in the world. The primary aim of HD treatment is not only to prolong survival but also to maintain and improve a patient’s quality of life.^[2]^ Previous studies have consistently reported that individuals undergoing hemodialysis experience a significantly lower quality of life compared with the general population.^[3,4]^ In patients receiving HD treatment, limitations in activities of daily living, sexual dysfunction, chronic fatigue, lack of social support, concerns about the future, economic difficulties, and dependence on healthcare professionals and caregivers adversely affect family and social life.^[5]^ Additionally, in HD patients, fluid restriction, lifestyle changes related to nutrition, multiple medication use, and the requirement to attend HD sessions 3 to 4 times a week impose significant physiological, psychological, and economic burdens, further compromising quality of life.^[6]^ Therefore, improving treatment adherence and promoting healthy lifestyle behaviors through structured, theory-based interventions may play a key role in enhancing the quality of life of patients undergoing hemodialysis.^[5]^ In order to achieve meaningful and sustainable results, patient education must be structured on clearly defined, theory-based frameworks. Effective education and counseling processes require the identification of not only health risks but also health-promoting factors; theory-based models enable these elements to be addressed in a systematic and holistic manner.^[7]^

PRECEDE–PROCEED model (PPM), developed by Green and Kreuter, provides a comprehensive theoretical framework for planning, implementing, and evaluating health promotion interventions.^[8]^ The updated PPM conceptualizes health promotion as a multistage process encompassing social, epidemiological, behavioral and environmental, educational and ecological, and administrative and policy assessments, followed by process, impact, and outcome evaluations.^[9]^ While the PRECEDE component focuses on identifying modifiable behavioral determinants and educational needs, the PROCEED component addresses policy, organizational, and environmental factors influencing behavior change from a holistic perspective. Grounded in social and behavioral sciences, epidemiology, and education, the PPM adopts a multidimensional approach that facilitates the development of targeted and patient-centered interventions.^[10–13]^

Numerous studies have demonstrated the effectiveness of the PPM across diverse age groups and patient populations. PPM-based educational interventions have been shown to improve the quality of life of patients with type 2 diabetes,^[11]^ enhance disease-related knowledge, treatment adherence, and blood pressure control in patients with hypertension,^[12]^ and increase physical activity levels among older adults.^[13]^ In addition, PPM has been successfully used to improve occupational health behaviors among nurses^[14]^ and oral health behaviors in school-aged children.^[15]^

Despite this growing evidence, studies applying PPM to hemodialysis patients remain limited. In Iran, Mosavi and colleagues reported that in hemodialysis patients trained using the PPM framework, weight gain between dialysis sessions, thirst levels, and self-management behaviors affected fluid intake.^[16]^ Similarly, Wahyuni et al demonstrated that PPM-based education improved the quality of life and self-efficacy in this population by reducing interdialytic weight gain.^[17]^ To our knowledge, no PPM-based intervention studies have been conducted among hemodialysis patients in Turkey. Therefore, the present study aimed primarily to evaluate the effects of a PPM-based educational program on health-promoting lifestyle behaviors in patients undergoing hemodialysis, while also examining treatment adherence and quality of life outcomes. This study addressed the following research hypotheses:

### 1.1. Research hypotheses

H0. A PPM-based education program does not improve treatment adherence (medication management, fluid restriction, and dietary adherence), healthy lifestyle behaviors, or quality of life in patients undergoing hemodialysis.

H1. A PPM-based education program improves treatment adherence (medication management, fluid restriction, and dietary adherence), healthy lifestyle behaviors, and quality of life in patients undergoing hemodialysis.

## 2. Methods

### 2.1. Study design

**Figure 1.**
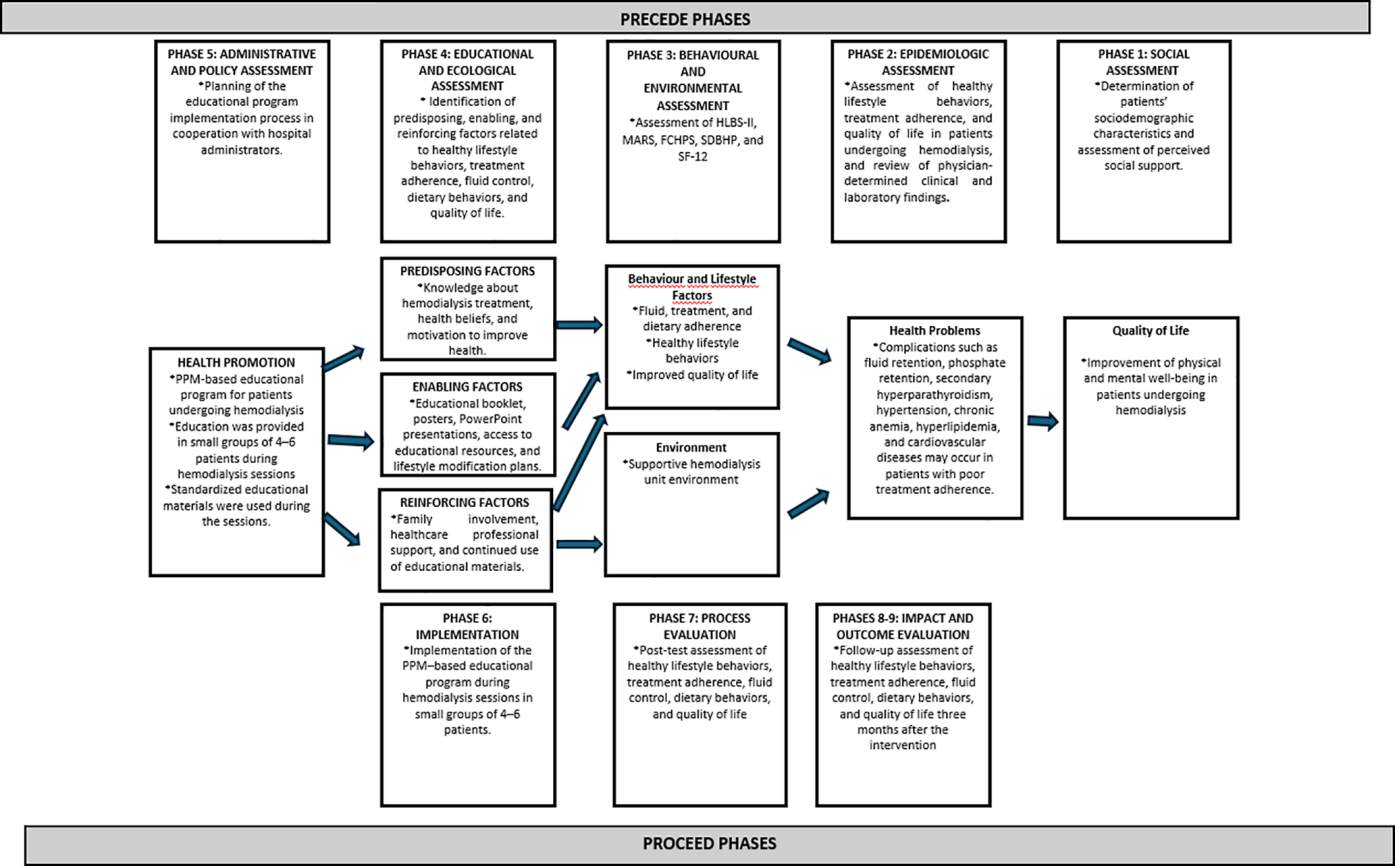
Application of the PRECEDE–PROCEED model-based education program in patients undergoing hemodialysis. Arrows indicate the sequential flow of the PRECEDE–PROCEED phases and the progression of the educational intervention. FCHPS = fluid control in hemodialysis patients scale, HLBS-II = healthy lifestyle behaviors scale II, MARS = medical treatment adherence rate scale, PPM = PRECEDE–PROCEED model, SDBHP = scale for dietary behaviors in hemodialysis patients, SF-12 = short form-12 quality of life scale.

This study was designed as a shift-level randomized clinical trial with a pretest–posttest–follow-up structure. The study aimed to evaluate the effectiveness of a community-based health education program developed based on the PPM. The application of the PPM in the development and implementation of the study is illustrated in Figure 1. The study was conducted in accordance with the CONSORT 2010 guidelines for randomized trials and was registered at ClinicalTrials.gov (NCT05955703).

### 2.2. Samples, participants, and randomization

This study was conducted at a single hemodialysis unit in Kars, Turkey. The sample consisted of purposively selected adult patients undergoing maintenance hemodialysis. Eligible patients were aged 18 years or older, had been receiving hemodialysis treatment for at least 6 months, scored ≥ 1 on the dialysis diet and fluid nonadherence questionnaire, scored between 1 and 7 on the medical treatment adherence rate scale (MARS), were oriented to person, place, and time, and had no psychiatric illness or condition that could impair written or verbal communication.

Although multiple outcome variables were evaluated in this study, healthy lifestyle behaviors measured using the healthy lifestyle behaviors scale II (HLBS-II) were identified as the primary outcome because the PPM primarily aims to improve positive health behaviors. The other scales related to treatment adherence and quality of life were evaluated as secondary outcomes.

The study’s sample size was calculated prior to data collection using GPower software (version 3.1.9.2). As no comparable reference study was available, Cohen standardized effect size was applied.^[18]^ Based on an effect size of 0.25, a significance level of 0.05, and a statistical power of 80%, the minimum required sample size was calculated as 64 patients, with 32 patients allocated to each group. To account for an anticipated 10% loss to follow-up, a total of 72 patients were planned for recruitment. The sample size estimation was structured to detect changes over time between groups in the primary outcome.

To minimize contamination between participants receiving hemodialysis during the same treatment sessions, randomization was performed at the hemodialysis schedule level rather than at the individual patient level. Thus, the unit of observation was the individual participant, whereas the unit of randomization was the dialysis schedule. The Monday–Wednesday–Friday and Tuesday–Thursday–Saturday dialysis schedules were assigned to either the intervention or control group using a simple lottery method conducted by a research staff member who was not involved in participant recruitment, intervention delivery, or outcome assessment. Patients were subsequently enrolled according to the group assignment of their existing dialysis schedule.

CONSORT flow diagram of participant recruitment, allocation, follow-up, and analysis. During the recruitment period, a total of 92 patients were assessed for eligibility. Before allocation, 20 patients were excluded, including 18 patients who were transferred to another dialysis center and 2 patients who did not meet the inclusion criteria. To minimize contamination between participants receiving hemodialysis during the same treatment sessions, allocation was performed according to hemodialysis treatment schedules/shifts. Hemodialysis schedules/shifts were assigned to either the intervention or control condition using a simple lottery method. Consequently, 72 patients were allocated to the intervention and control groups according to their existing dialysis schedules, with 36 patients in each group. During the follow-up period, 2 patients in the intervention group did not complete the educational program, while 4 patients in the control group were transferred to another dialysis center due to relocation. As a result, the study was completed with 66 patients, including 34 patients in the intervention group and 32 patients in the control group.

### 2.3. Blinding

Due to the nature of the educational intervention, blinding of the patients and researchers was not feasible. However, statistical analyses were conducted by a statistician who was blinded to the group allocation of the patients.

### 2.4. Data collection instruments

Data were collected by the researcher at the hemodialysis unit. The study’s data were collected using the Patient Information Form, MARS, fluid control in hemodialysis patients scale (FCHPS), scale for dietary behaviours in hemodialysis patients (SDBHP), HLBS-II, and short form-12 quality of life scale (SF-12).

#### 2.4.1. Patient information form

The form was prepared using the literature data and consisted of 12 questions to determine the sociodemographic and clinical characteristics of the patients.^[16,17]^

#### 2.4.2. MARS

The MARS was developed by Thompson as a brief and easy-to-administer instrument for assessing treatment adherence.^[19]^ The Turkish validity and reliability of the scale were evaluated within the scope of a specialty thesis conducted among patients with chronic psychosis.^[20]^ Additionally, it was observed that the Turkish adaptation of MARS was used in studies evaluating medication adherence among individuals with chronic diseases.^[21]^

The scale consists of 10 items with yes/no response options. Higher scores indicate better medication adherence, whereas lower scores reflect nonadherence. The scale was used in the present study because it is short and easy to understand. In the Turkish adaptation conducted by Koç, the scale demonstrated satisfactory internal consistency, with a Cronbach α coefficient of 0.92.^[20]^ In the present study, the Cronbach α reliability coefficient was 0.90.

#### 2.4.3. FCHPS

The FCHPS was developed by Albayrak Coşar and Çinar Pakyüz to assess the knowledge, behaviors, and attitudes related to fluid restriction in patients undergoing hemodialysis.^[22]^ The scale consists of 24 items organized into 3 subscales: knowledge (items 1–7), behavior (items 8–18), and attitude (items 19–24). Items are rated on a three-point Likert scale, with higher total scores indicating better adherence to fluid control. The Turkish version of the scale demonstrated good internal consistency, with a Cronbach α coefficient of 0.88. In the present study, the Cronbach α reliability coefficient of the scale was 0.79.

#### 2.4.4. SDBHP

The SDBHP was developed by Düzalan and Pakyüz to assess dietary behaviors and attitudes related to diet and fluid restriction in patients undergoing hemodialysis.^[23]^ The scale consists of 13 items rated on a five-point Likert scale, with higher scores indicating better dietary adherence. The scale has a single-factor structure and contains no reverse-scored items. The original scale demonstrated acceptable internal consistency, with a Cronbach α coefficient of 0.73. In the present study, the Cronbach α reliability coefficient was 0.86.

#### 2.4.5. HLBS-II

The HLBS-II scale was originally developed by Walker, Sechrist, and Pender to assess health-promoting behaviors associated with a healthy lifestyle.^[24]^ The revised version of the scale, in which the subscales were reorganized to improve internal consistency, was adapted into Turkish, and its validity and reliability were confirmed by Bahar et al.^[25]^ The scale consists of 52 items and 6 subscales, with higher total scores indicating more frequent engagement in health-promoting behaviors. The scale has been widely used to determine health promotion behaviors and to evaluate the effectiveness of health promotion programs. The Turkish version of the scale demonstrated good internal consistency, with a Cronbach α coefficient of 0.92. In the present study, the Cronbach α reliability coefficient was 0.95.

#### 2.4.6. SF-12

The SF-12 is a brief instrument derived from the SF-36 Health Survey, consisting of 12 items designed to assess physical and mental health components. The scale covers the domains of physical component and mental component. The Turkish validity and reliability study of the scale was conducted by Soylu and Kütük, who reported satisfactory internal consistency coefficients for the component summary scores (Cronbach α = 0.73 for the physical component and α = 0.72 for the mental component).^[26]^ In the present study, the Cronbach α reliability coefficients were 0.62 for the physical component summary and 0.63 for the mental component summary.

### 2.5. Intervention

#### 2.5.1. Stage 1: social assessment

In this phase, the factors influencing treatment adherence, healthy lifestyle behaviors, and the quality of life of patients undergoing HD were assessed using multiple data collection methods, including a comprehensive literature review and structured individual interviews. The pretest results revealed low levels of treatment adherence, healthy lifestyle behaviors, and quality of life in patients undergoing hemodialysis.

#### 2.5.2. Stages 2 and 3: epidemiological assessment and behavioral–environmental assessment

In this phase, the researchers systematically collected data on the treatment adherence, healthy lifestyle behaviors, and quality of life of patients undergoing hemodialysis. Data were obtained through an extensive review of national and international literature using online databases and other relevant scientific sources. The behavioral and non-behavioral factors influencing these outcomes were identified and classified within the PRECEDE framework.

#### 2.5.3. Stage 4: educational and ecological assessment

In the educational and ecological assessment phase of the PPM, predisposing, enabling, and reinforcing factors influencing health-related behaviors were identified through interviews with patients undergoing HD, their family members, and healthcare professionals working in the HD unit. Based on the literature, knowledge, attitudes, and awareness were defined as the main predisposing factors, and these factors were assessed using the MARS, FCHPS, and SDBHP instruments.

The identified enabling factors were operationalized as structural and contextual conditions designed to facilitate the patients’ and family members’ access to and participation in the educational program. The identified reinforcing factors were incorporated into the intervention through standardized educational materials (educational booklet, posters, and PowerPoint presentations), family involvement, and verbal encouragement provided by healthcare professionals. These enabling and reinforcing factors were not measured quantitatively; rather, they were intentionally embedded within the intervention to support and sustain behavior change in accordance with the theoretical framework of the model. The educational booklet served as the primary enabling and reinforcing resource, and all educational materials contained identical content to ensure consistency.

The quality of the educational booklet was evaluated using the DISCERN instrument,^[27]^ yielding a mean score of 69.92 ± 5.36. Readability was assessed using the Flesch Reading Ease Index, which was adapted to Turkish^[28,29]^ with a score of 76.42, indicating easy readability and suitability for the target HD population.

#### 2.5.4. Stage 5: assessment of management and policies and designing interventions

In this stage, discussions were conducted with the hospital administrators at the site where the training programme was being implemented, and the necessary planning for the implementation of the programme was completed.

#### 2.5.5. Stage 6: implementation

The educational program was implemented over an eight-week period during hemodialysis sessions, with 15 to 20 minutes sessions conducted in small groups of 4 to 6 patients. To ensure consistency and fidelity of the intervention, all sessions were delivered by the same researcher. The topics included renal function and CKD, symptom management and treatment, fluid and nutritional management, medication use and interpretation of laboratory findings, common clinical problems with potential solutions, and evidence-based recommendations for maintaining a healthy lifestyle.

#### 2.5.6. Stage 7: assessment of process

During the implementation of the educational program, the progress of the patients was systematically monitored through regular assessments and a posttest, in alignment with the predetermined objectives.

#### 2.5.7. Stages 8 and 9: assessment of the impact and outcome

Follow-up assessments were conducted 3 months after the intervention. Patients in the control group received routine healthcare during the study period, and no educational intervention was administered. Upon completion of the follow-up assessments, the control group patients were provided with a single educational session along with the corresponding booklets.

### 2.6. Ethical principles

Ethical approval for this study was obtained from the Atatürk University Faculty of Nursing Ethics Committee (date: September 15, 2021; approval number: 2021-4/13). The study was conducted in accordance with the principles of the Declaration of Helsinki. Written institutional permission was obtained from the HD units where the study was conducted. All patients were informed about the study, and written informed consent was obtained prior to their participation.

### 2.7. Data analysis

Study data were analyzed using IBM SPSS Statistics version 27.0 (IBM Corp., Armonk). Categorical variables were presented as numbers and percentages, whereas continuous variables were expressed as mean ± standard deviation. Baseline group comparisons were performed using the Pearson chi-square test, Fisher exact test, or Student *t* test, as appropriate. Normality was assessed using skewness coefficients.

Primary and secondary outcome variables were analyzed using linear mixed-effects models. Group (intervention/control), time (baseline, post-test, and follow-up), and the group × time interaction were included as fixed effects, whereas participants were specified as random effects. Results were reported using *F* values, *p* values, and partial eta-squared (η*p*^2^) effect sizes. Because only 2 dialysis schedule groups were available, analyses were conducted at the individual participant level, and clustering by schedule was not included in the statistical model.

When significant group × time interactions were identified, further analyses were performed. Between-group differences at each time point were evaluated using independent-samples *t*-tests, whereas within-group changes over time were assessed using paired-samples *t*-tests with Bonferroni correction. Cohen *d* effect sizes and 95% confidence intervals were also calculated. Participants with incomplete follow-up data were excluded from the analyses, and complete-case analysis was performed. Statistical significance was set at *P* < .05.

## 3. Results

The results of the study are presented below. The study was conducted with a total of 66 HD patients, 34 in the intervention group and 32 in the control group. In the chi-square test, no significant difference was found between the 2 groups in the sample in terms of their sociodemographic and clinical characteristics (*P* > .05). The results showed that both groups were similar and homogeneous in terms of descriptive characteristics (Table 1).

**Table 1:** Baseline characteristics of the intervention and control groups.

Descriptive characteristics	Intervention group (n = 34)		Control group (n = 32)		Test and significance
	n	%	n	%	
Age					
<50	8	23.5	6	18.8	χ^2=^1.487* P = .475
51–65	17	50.0	13	40.6	
≥66	9	26.5	13	40.6	
Sex					
Female	11	32.4	14	43.8	χ^2^ = 0.910** P = .447
Male	23	67.6	18	56.2	
Education status					
Illiterate	2	5.9	2	6.2	χ^2^ = 5.551* P = .235
Literate	6	17.6	3	9.4	
Primary school	14	41.2	17	53.1	
Middle school	12	35.3	7	21.9	
Higher education	0	0.0	3	9.4	
Marital status					
Married	30	88.2	29	90.6	χ^2^ = 0.099** P = .753
Single	4	11.8	3	9.4	
Employment status					
Employed	2	5.9	2	6.2	χ^2^ = 0.011** P = 1.000
Unemployed	32	94.1	30	93.8	
Income status					
Income less than expenses	12	35.3	9	28.1	χ^2^ = 1.394* P = .498
Income equal to expenses	21	61.8	20	62.5	
Income more than expenses	1	2.9	3	9.4	
Smoking status					
Yes	2	5.9	2	6.2	χ^2^ = 0.004** P = .950
No	32	94.1	30	93.8	
Alcohol consumption					
Yes	0	0.0	3	9.4	χ^2^ = 3.339** P = .108
No	34	100.0	29	90.6	
Comorbidity					
Yes	27	79.4	27	84.4	χ^2^ = 0.273** P = .752
No	7	20.6	5	15.6	
Previous hemodialysis education					
Yes	12	35.3	9	28.1	χ^2^ = 0.391** P = .603
No	22	64.7	23	71.9	
Duration of disease	S	*X* ± SD	S	*X* ± SD	*t* = 0.102*** P = .919
	34	9.14 ± 5.51	32	9.00 ± 6.20	
Duration of hemodialysis	34	7.35 ± 5.90	32	6.09 ± 5.74	*t* = 0.873*** P = .386

* Pearson chi-square test.

** Fisher exact test.

*** Independent samples *t*-test. Values are presented as n (%) or mean ± standard deviation (SD).

According to the results presented in Table 2, a statistically significant increase in MARS scores was observed over time in favor of the intervention group. While the intervention group demonstrated significant increases from baseline to post-test and follow-up assessments, no significant change was observed in the control group. Significant group, time, and group × time interaction effects were identified for MARS scores (Table 2). In addition, effect size analyses demonstrated large intervention effects. These findings suggest that the intervention contributed to improvements in medication adherence over time.

**Table 2:** Group, time, and group × time interaction effects on MARS, FCHPS, and SDBHP scores.

Scale/ subscales	Time	Intervention group *X* ± SD	Control group *X* ± SD	Group difference [95% CI]	*p* †	*d*	*F*(*g*)	*p*(g)	*F*(*z*)	*p*(*z*)	*F*(*g* × *z*)	*p*(*g* × *z*)	η*p*^2^
MARS	Pretest	4.35 ± 2.35	4.34 ± 2.39	0.01 [−1.13, 1.15]	.987 ns	0.00	9.70	**.002**	71.06	**<.001**	67.35	**<.001**	0.513
	Posttest	9.59 ± 0.66(***)	4.25 ± 2.26 (ns)	5.34 [4.53, 6.15]	**<.001** ***	3.25							
	Follow-up	9.26 ± 0.83 (***)	4.44 ± 2.29 (ns)	4.83 [3.99, 5.67]	**<.001** ***	2.84							
FCHPS-knowledge	Pretest	19.18 ± 2.41	17.81 ± 2.25	1.36 [0.24, 2.49]	**.021** *	0.59	3.29	.072	8.11	**<.001**	0.55	.579	0.009
	Posttest	19.00 ± 0.85 (ns)	17.84 ± 2.28 (ns)	1.16 [0.31, 2.01]	**.007** **	0.69							
	Follow-up	18.24 ± 1.05 (*)	16.47 ± 2.49 (*)	1.77 [0.84, 2.70]	**<.001** ***	0.94							
FCHPS-behavior	Pretest	19.18 ± 6.31	17.19 ± 4.43	1.99 [−0.63, 4.61]	.145 ns	0.36	12.86	**<.001**	36.41	**<.001**	40.94	**<.001**	0.390
	Posttest	28.76 ± 1.23 (***)	17.19 ± 4.43 (ns)	11.58 [9.99, 13.17]	**<.001** ***	3.61							
	Follow-up	28.82 ± 1.11 (***)	16.31 ± 3.76 (ns)	12.51 [11.15, 13.87]	**<.001** ***	4.57							
FCHPS-attitude	Pretest	6.56 ± 1.83	6.94 ± 1.70	−0.38 [−1.23, 0.47]	.388 ns	−0.21	1.17	.282	224.09	**<.001**	470.80	**<.001**	0.880
	Posttest	13.50 ± 0.83 (***)	6.94 ± 1.70 (ns)	6.56 [5.91, 7.21]	**<.001** ***	4.95							
	Follow-up	15.72 ± 1.42 (***)	7.71 ± 0.84 (***)	8.01 [8.58, 7.45]	**<.001** ***	6.93							
FCHPS-total	Pretest	44.91 ± 8.09	41.94 ± 5.31	2.97 [−0.31, 6.26]	.084 ns	0.43	10.57	**<.001**	72.92	**<.001**	56.97	**<.001**	0.473
	Posttest	61.26 ± 1.85 (***)	41.97 ± 5.39 (ns)	19.30 [17.30, 21.29]	**<.001** ***	4.88							
	Follow-up	54.76 ± 1.83 (***)	48.50 ± 4.44 (***)	6.26 [4.61, 7.92]	**<.001** ***	1.87							
SDBHP	Pretest	31.00 ± 7.66	27.75 ± 5.99	3.25 [−0.06, 6.56]	.060 ns	0.47	40.14	**<.001**	137.18	**<.001**	119.83	**<.001**	0.652
	Posttest	51.68 ± 1.30 (***)	27.72 ± 6.00 (ns)	23.96 [21.84, 26.08]	**<.001** ***	5.60							
	Follow-up	52.97 ± 1.66 (***)	28.53 ± 4.10 (ns)	24.44 [22.91, 25.97]	**<.001** ***	7.90							

ns: *P* > .05.

*d* = Cohen *d*, FCHPS = fluid control in hemodialysis patients scale, MARS = medication adherence rating scale, SDBHP = scale for dietary behaviors in hemodialysis patient, ηp^2^ = partial eta squared.

† Independent samples *t*-test.

* *P* < .05.

** *P* < .01.

*** *P* < .001.

For the FCHPS scale, only the time effect was significant in the knowledge subscale, whereas the group × time interaction was not significant (Table 2). Significant group × time interactions were identified for the behavior, attitude, and total scores. In the behavior subscale, the intervention group demonstrated significant increases at both post-test and follow-up assessments. A similar pattern was observed for the attitude subscale and total score, with improvements in the intervention group and limited changes in the control group. Effect size analyses indicated moderate-to-large intervention effects (Table 2).

SDBHP scores increased significantly from baseline to post-test and follow-up assessments in the intervention group, whereas no significant change was observed in the control group. Significant group, time, and group × time interaction effects were identified for SDBHP scores (Table 2). In addition, effect size analyses demonstrated large intervention effects. These findings suggest that the intervention improved dietary behaviors over time in favor of the intervention group.

Table 3 presents the pretest, post-test, and follow-up findings for the primary outcome measure, HLBS-II, including its total and subscale scores according to the study groups.

**Table 3:** Group, time, and group × time interaction effects for the HLBS-II scale and its subscales.

Scale/ subscales	Time	Intervention group *X* ± SD	Control group *X* ± SD	Group difference [95% CI]	*p* †	*d*	*F*(*g*)	*p*(*g*)	*F*(*z*)	*p*(*z*)	*F*(*g* × *z*)	*p*(*g* × *z*)	η*p*^2^
Health responsibility	Pretest	18.94 ± 3.90	17.34 ± 3.43	1.60 [−0.17, 3.37]	.083 ns	0.43	28.49	**<.001**	106.34	**<.001**	77.47	**<.001**	0.548
	Posttest	28.24 ± 1.35 (***)	17.31 ± 3.40 (ns)	10.92 [9.66, 12.19]	**<.001** ***	4.27							
	Follow-up	29.82 ± 1.78 (***)	18.47 ± 2.64 (ns)	11.35 [10.26, 12.45]	**<.001** ***	5.07							
Physical activity	Pretest	13.62 ± 4.09	11.78 ± 3.97	1.84 [−0.11, 3.78]	.069 ns	0.46	18.89	**<.001**	46.29	**<.001**	34.59	**<.001**	0.351
	Posttest	21.35 ± 1.47 (***)	11.78 ± 3.97 (ns)	9.57 [8.11, 11.03]	**<.001** ***	3.23							
	Follow-up	22.35 ± 1.43 (***)	12.62 ± 3.63 (ns)	9.73 [8.38, 11.08]	**<.001** ***	3.56							
Nutrition	Pretest	19.97 ± 3.26	19.88 ± 3.41	0.10 [−1.51, 1.71]	.908 ns	0.03	0.97	.327	1.38	.256	3.55	**.032**	0.053
	Posttest	19.06 ± 1.18 (ns)	19.88 ± 3.41 (ns)	−0.82 [−2.06, 0.43]	.193 ns	−0.32							
	Follow-up	19.12 ± 1.12 (ns)	21.06 ± 2.56 (ns)	−1.94 [−2.91, −0.98]	**<.001** ***	−0.99							
Spiritual growth	Pretest	19.53 ± 3.57	18.06 ± 3.53	1.47 [−0.25, 3.18]	.098 ns	0.41	21.18	**<.001**	83.42	**<.001**	52.58	**<.001**	0.451
	Posttest	27.68 ± 1.72 (***)	18.09 ± 3.53 (ns)	9.58 [8.23, 10.94]	**<.001** ***	3.48							
	Follow-up	29.18 ± 1.73 (***)	19.56 ± 2.49 (ns)	9.61 [8.57, 10.65]	**<.001** ***	4.51							
Interpersonal relations	Pretest	19.74 ± 3.93	19.75 ± 3.69	−0.01 [−1.85, 1.82]	.988 ns	−0.00	10.20	**.002**	61.40	**<.001**	53.34	**<.001**	0.455
	Posttest	27.29 ± 1.31 (***)	19.75 ± 3.69 (ns)	7.54 [6.19, 8.90]	**<.001** ***	2.76							
	Follow-up	28.44 ± 1.73 (***)	20.03 ± 3.24 (ns)	8.41 [7.15, 9.67]	**<.001** ***	3.27							
Stress management	Pretest	15.82 ± 3.08	14.81 ± 3.15	1.01 [−0.49, 2.51]	.192 ns	0.32	29.26	**<.001**	92.28	**<.001**	64.93	**<.001**	0.504
	Posttest	23.59 ± 1.76 (***)	14.81 ± 3.15 (ns)	8.78 [7.54, 10.02]	**<.001** ***	3.47							
	Follow-up	25.32 ± 1.63 (***)	15.84 ± 2.68 (ns)	9.48 [8.40, 10.56]	**<.001** ***	4.31							
HLBS-II total	Pretest	107.62 ± 18.73	101.62 ± 18.12	5.99 [−2.90, 14.88]	.192 ns	0.33	19.69	**<.001**	82.86	**<.001**	50.55	**<.001**	0.441
	Posttest	137.91 ± 5.47 (***)	101.66 ± 18.13 (ns)	36.26 [29.71, 42.80]	**<.001** ***	2.74							
	Follow-up	154.24 ± 3.92 (***)	107.59 ± 14.04 (ns)	46.64 [41.60, 51.68]	**<.001** ***	4.59							

ns: *P* > .05.

*d* = Cohen *d*, HLBS-II = healthy lifestyle behaviors scale, η*p*^2^ = partial eta squared.

† Independent samples *t*-test.

* *P* < .05.

** *P* < .01.

*** *P* < .001.

When the HLBS-II total score and its subscales were examined, statistically significant increases over time were observed in the intervention group for health responsibility, physical activity, spiritual growth, interpersonal relations, stress management, and total HLBS-II scores. In contrast, these changes remained limited in the control group. Significant group × time interaction effects were identified for these subscales (all *P* < .001, Table 3). Significant group × time interaction effects were also identified for total HLBS-II scores (*P* < .001, η*p*^2^ = 0.44, Table 3), indicating greater improvements over time in the intervention group compared with the control group. Effect size analyses indicated moderate-to-large intervention effects, particularly for health responsibility, stress management, and total HLBS-II scores. These findings suggest that the intervention had a positive effect on health-promoting lifestyle behaviors.

For the nutrition subscale, the main effects of group and time were not statistically significant; however, the group × time interaction was significant (*P* = .032, η*p*^2^ = 0.05, Table 3). This finding suggests that changes in nutritional behaviors differed between groups, although the relatively small effect size indicates that the magnitude of this change was limited.Table 4 presents the analysis results for the SF-12 physical and mental component scores used to evaluate the effects of the intervention program on patients’ perceived quality of life.

**Table 4:** Results of group, time, and group × time interaction analyses for the SF-12 quality of life scale.

Scale/ subscales	Time	Intervention group *X* ± SD	Control group *X* ± SD	Group difference [95% CI]	*p* †	*d*	*F*(*g*)	*p*(*g*)	*F*(*z*)	*p*(*z*)	*F*(*g* × *z*)	*p*(*g* × *z*)	η*p*^2^
Physical component	Pretest	26.18 ± 3.38	26.67 ± 3.36	−0.49 [−2.11, 1.14]	.558 ns	−0.14	30.52	**<.001**	133.87	**<.001**	96.80	**<.001**	0.602
	Posttest	39.12 ± 2.81 (***)	26.67 ± 3.36 (ns)	12.45 [10.95, 13.95]	**<.001** ***	4.03							
	Follow-up	42.91 ± 4.63 (***)	28.20 ± 4.14 (ns)	14.71 [12.59, 16.82]	**<.001** ***	3.34							
Mental component	Pretest	31.20 ± 5.97	34.19 ± 7.73	−2.99 [−6.34, 0.36]	.083 ns	−0.43	8.20	**.005**	53.82	**<.001**	25.14	**<.001**	0.284
	Posttest	40.88 ± 2.10 (***)	34.04 ± 7.82 (ns)	6.84 [4.00, 9.68]	**<.001** ***	1.22							
	Follow-up	45.94 ± 4.44 (***)	37.30 ± 5.60 (ns)	8.64 [6.20, 11.09]	**<.001** ***	1.72							

ns: *P* > .05.

*d* = Cohen *d*, η*p*^2^ = partial eta squared.

† Independent samples *t*-test.

* *P* < .05.

** *P* < .01.

*** *P* < .001.

Evaluation of the SF-12 quality of life scale demonstrated statistically significant increases in both physical and mental component scores at post-test and follow-up assessments in the intervention group, whereas scores in the control group remained close to baseline levels (Table 4).

According to the analysis results, significant group × time interaction effects were identified for both the physical and mental component scores (Table 4). Effect size analyses indicated large intervention effects, particularly for the physical component score. In addition, between-group comparisons demonstrated large effect sizes favoring the intervention group at both post-test and follow-up assessments. These findings suggest that the intervention positively influenced perceived quality of life over time.

## 4. Discussion

Failure to achieve treatment compliance in HD patients leads to various problems such as increased mortality, morbidity, and health costs.^[30]^ In HD patients, treatment compliance is assessed through compliance with medical treatment, compliance with fluid restriction, and compliance with a dietary program.^[31]^ It is known that the treatment compliance of patients is generally low; however, education sessions aimed at behavioral change have been shown to improve treatment compliance in HD patients, to contribute positively to quality of life, and to promote healthy lifestyle behaviors.^[4,5,32]^

In this study, PPM-based education resulted in significantly higher mean MARS and SDBHP scores in the intervention group compared to the control group at both posttest and follow-up assessments. These findings suggest that interventions targeting predisposing factors, particularly the knowledge, attitudes, and awareness of patients regarding medication use and dietary management, may positively influence self-reported treatment adherence behaviors. Consistent with these results, Griva et al reported that educational interventions focusing on medication management, dietary control, and fluid restriction improved adherence behaviors among HD patients.^[33]^ Similarly, Cho demonstrated that education aimed at modifying behavioral and predisposing factors related to medication, diet, and fluid management led to improved overall treatment compliance in this population.^[34]^

PPM-based education was associated with a significant improvement in fluid restriction compliance, as evidenced by higher FCHPS scores in the intervention group compared with the control group at the posttest and follow-up assessments. These findings indicate that educational interventions grounded in the PPM may facilitate behavior change related to fluid management in patients undergoing HD. Consistent with these findings, Wahyuni et al reported that interdialytic weight gain was associated with thirst perception, self-management, and fluid intake behaviors among hemodialysis patients using a PPM-based approach.^[17]^ In addition, previous studies using alternative educational models similarly demonstrated improvements in adherence to fluid restriction in this population.^[35]^ Although no significant improvement in knowledge scores was observed following the intervention, knowledge scores were relatively high at baseline in both groups. This finding suggests that knowledge alone may be insufficient to achieve sustained behavioral change and underscores the importance of repeated, behavior-focused educational strategies with extended follow-up. Nevertheless, fluctuations observed in the attitude subscale scores at follow-up suggest that attitudinal changes may require sustained educational reinforcement to maintain long-term behavioral change.^[36]^

In this study, the identified enabling and reinforcing factors were not evaluated as quantitative variables but were rather integrated into the intervention in line with the PPM. Standardized educational materials, family involvement, and verbal encouragement provided by healthcare professionals were used to support participation in the educational process and to reinforce behavior change. Although the specific contribution of these factors in HD could not be determined quantitatively, they may have played an indirect role in the observed improvements in healthy lifestyle behaviors and quality of life.^[37,38]^

The primary outcome of this study, health-promoting lifestyle behaviors measured using the HLBS-II, showed significant improvement in the intervention group compared with the control group at both posttest and follow-up assessments. However, no significant change was observed in the nutrition subscale at posttest. This finding is more likely attributable to the characteristics of the measurement instrument rather than to the ineffectiveness of the intervention. The nutrition subscale of the HLBS-II was originally developed for healthy populations and may not adequately capture the dietary restrictions and nutritional challenges specific to patients undergoing hemodialysis. Consistent with previous reports indicating persistently low nutrition subscale scores among HD patients,^[39,40]^ this result may be considered a measurement-related limitation of the study. Nevertheless, previous studies have demonstrated that PPM-based educational interventions positively influenced healthy lifestyle behaviors, self-care, and awareness across different patient populations.^[12,16,41]^ In the present study, the observed improvement in health-promoting lifestyle behaviors is clinically meaningful, particularly given the substantial physiological and psychosocial burden experienced by patients receiving HD, which is known to adversely affect their quality of life.

In this study, the PPM-based educational intervention was associated with higher self-reported quality-of-life scores, as reflected by higher SF-12 subscale scores in the intervention group at both the posttest and follow-up assessments. Similar improvements in quality of life following PPM-based education have been reported among HD patients^[16]^ as well as in other populations, including elderly individuals, patients with asthma, and patients with diabetes mellitus.^[42–44]^ However, some studies have reported no statistically significant between-group differences despite increases in mean quality of life scores of HD patients, suggesting that improvements in quality of life may require longer periods to become evident.^[45,46]^ Quality of life is a multidimensional and dynamic outcome that often reflects the cumulative effects of sustained behavioral change over time. Therefore, differences in follow-up duration across studies may partly explain the variability in findings, and longer follow-up periods may be necessary to fully capture the long-term impact of PPM-based educational interventions.

Overall, the findings of this study suggest that PPM-based educational interventions may contribute to improvements in self-reported health-promoting lifestyle behaviors, adherence-related behaviors, and self-reported quality of life among patients undergoing hemodialysis. Given the central role of nurses in patient education and behavioral counseling, the integration of theory-based educational approaches such as the PPM into routine hemodialysis care may help support long-term behavioral change and patient well-being.

## 5. Conclusion

This study suggested that a PPM-based educational program may be associated with improvements in self-reported health-promoting lifestyle behaviors, adherence-related behaviors, and self-reported quality of life among patients undergoing hemodialysis. Improvements were observed both immediately after the intervention and at the three-month follow-up assessment, suggesting that the effects of the intervention may be sustained over time. The findings support the potential clinical applicability of the PPM as a theory-based framework for patient education in hemodialysis care. Integrating PPM-based educational interventions into routine clinical practice may assist nephrology nurses and case managers in providing structured and individualized patient education. However, the findings should be interpreted cautiously, as the outcomes were assessed using self-reported instruments, and objective dialysis-related indicators were not included in the study. Future studies with larger sample sizes, longer follow-up periods, objective dialysis-related outcome measures, and more rigorous allocation procedures are needed to confirm these findings and improve the generalizability of the results.

### 5.1. Limitations

The relatively small sample size and conducting the study in a single center within one province may limit the generalizability of the findings. In addition, neither the researchers nor the participants were blinded, which may have introduced bias into the study. The follow-up period was limited to 3 months after the intervention, restricting the evaluation of long-term effects.

Healthy lifestyle behaviors were assessed using the HLBS-II scale, and no significant changes were observed in the nutrition subscale. This finding may be related to the limited ability of the nutrition subscale to fully capture dietary practices and restrictions specific to patients undergoing hemodialysis.

Furthermore, the identified enabling and reinforcing factors within the PPM were incorporated as part of the intervention but were not quantitatively measured. Therefore, the specific contribution of these factors to the outcome variables could not be directly assessed.

A limitation of this study is that an intention-to-treat analysis was not performed. Participants with incomplete follow-up data were excluded from the analyses, which may have increased the risk of attrition bias despite the preservation of baseline group homogeneity.

Additionally, the relatively lower internal consistency coefficients observed for the SF-12 in this sample should be considered when interpreting the quality-of-life findings. Another limitation is that participant allocation was based on dialysis schedules. As only 2 dialysis schedules were included in the study, clustering effects could not be evaluated separately.

## Acknowledgments

We would like to thank all dialysis center staff and hemodialysis patients who participated in the study. This manuscript was produced from a doctoral thesis (Sariaslan A., Kavurmaci M., Evaluation of the Effect of the Training Program based on the Precede-Proceed Model on Adherence to Treatment and Healthy Lifestyle Behaviors of Hemodialysis Patients, Atatürk University, Institute of Health Sciences, 2022).

## Author contributions

**Conceptualization:** Ayşenur Sariaslan, Mehtap Kavurmaci.

**Data curation:** Ayşenur Sariaslan, Mehtap Kavurmaci.

**Investigation:** Ayşenur Sariaslan.

**Methodology:** Ayşenur Sariaslan, Mehtap Kavurmaci.

**Project administration:** Mehtap Kavurmaci.

**Resources:** Ayşenur Sariaslan.

**Supervision:** Mehtap Kavurmaci.

**Writing – original draft:** Ayşenur Sariaslan.

**Writing – review & editing:** Ayşenur Sariaslan, Mehtap Kavurmaci.
